# Improving long-term multivariate time series forecasting with a seasonal-trend decomposition-based 2-dimensional temporal convolution dense network

**DOI:** 10.1038/s41598-024-52240-y

**Published:** 2024-01-19

**Authors:** Jianhua Hao, Fangai Liu

**Affiliations:** School of Information Science and Engineering, Shandong Normal University, Jinan, 250358 Shandong China

**Keywords:** Information technology, Scientific data, Statistics

## Abstract

Improving the accuracy of long-term multivariate time series forecasting is important for practical applications. Various Transformer-based solutions emerging for time series forecasting. Recently, some studies have verified that the most Transformer-based methods are outperformed by simple linear models in long-term multivariate time series forecasting. However, these methods have some limitations in exploring complex interdependencies among various subseries in multivariate time series. They also fall short in leveraging the temporal features of the data sequences effectively, such as seasonality and trends. In this study, we propose a novel seasonal-trend decomposition-based 2-dimensional temporal convolution dense network (STL-2DTCDN) to deal with these issues. We incorporate the seasonal-trend decomposition based on loess (STL) to explore the trend and seasonal features of the original data. Particularly, a 2-dimensional temporal convolution dense network (2DTCDN) is designed to capture complex interdependencies among various time series in multivariate time series. To evaluate our approach, we conduct experiments on six datasets. The results demonstrate that STL-2DTCDN outperforms existing methods in long-term multivariate time series forecasting.

## Introduction

Long-term series forecasting of multivariate time series has already played a significant role in numerous practical fields, such as transportation^1^, meteorology^2^, energy management^3^, finance^4^, environment^5^, etc. In these practical application scenarios, we can explore a mass of historical data to forecast the future value for making decisions and planning in advance. The task of time series prediction is divided into multivariate and univariate based on the number of temporal variables involved. Multivariate time series forecasting tasks holds extremely challenges when dealing with long-term setting, yet they hold crucial practical significance. Hence, we are dedicated to developing appropriate methods to improve the forecasting performance of models in long-term multivariate time series.

Many scholars have proposed various methods for time series forecasting. Traditional statistic-based methods are mainly applied in univariate time series forecasting tasks, for example, Autoregressive (AR)^6^, Autoregressive Integrated Moving Average (ARIMA)^7^, Exponential Smoothing (ES)^8^, and more. However, these traditional methods encounter challenges in capturing intricate nonlinear dependencies within long-term multivariate time series. For the past few years, deep learning has made great progress in the field of time series forecasting. Recurrent Neural Network (RNN) is an important model in the area of sequence modeling and are widely used in natural language processing^9^. Given the sequential nature of time series data, numerous RNN-based models and their variants are employed for time series forecasting^10–13^. Furthermore, Convolutional Neural Network (CNN) and its variants, such as Temporal Convolutional Network (TCN), are combined with RNN to enhance the model's capability in capturing local temporal features^14,15^. Additionally, to enhance the robustness and reliability of forecasting results, some works propose ensemble models for prediction^16–18^^.^

In the past few years, Transformer^19^ has demonstrated remarkable effectiveness in the domains of picture processing^20^ and language processing^21^. Transformer-based models exhibit superior performance in exploring long-term dependencies compared to RNN models. Therefore, numerous works have designed various prediction methods with the Transformer architecture. Nevertheless, the quadratic complexity of the calculating self-attention in both memory and time has been the limitation of applying Transformer to time series forecasting problem in long-term. Some studies meticulously design effective modules to address these challenges. By combining causal convolution with the Transformer architecture, the Logtran^22^ introduces a novel attention mechanism called LogSparse self-attention. In this innovative method, the queries and keys for self-attention are generated through causal convolution. Reformer^23^ replace self-attention with a novel locality-sensitive hashing and uses reversible residual to replace the standard residual. Informer^24^ introduces the ProbSparse self-attention mechanism, focusing on extracting important queries. Autoformer^25^ designs the auto-correlation and combines the sequence decomposition with Transformer architecture. Fedformer^26^ applies Fourier to enhance the performance of Transformer. PatchTST^27^ uses patches of time series data as tokens for the Transformer. CNformer^28^ proposes a CNN-based encoder-decoder attention mechanism to replace the vanilla attention mechanism. Triformer^29^ presents a triangular structure with the Patch Attention. While Transformer-based solutions have made great success in long-term time series forecasting, recent studies have indicated that most of Transformer-based methods can be outperformed by simple linear models. For instance, Zeng et al.^30^ introduce a simple linear model and achieve remarkable performance on forecasting benchmarks. Das et al.^31^ design a novel Time-series Dense Encoder (TiDE) model to solve time series prediction tasks in long-term, which can explore non-linear dependencies and enjoys the speed of linear models.

Although various Transformer-based models and linear models have made valuable contributions to time series forecasting, they often fall short when capturing complex interdependencies among components in long-term. Additionally, they have not effectively leveraged the temporal characteristics of the data sequences, such as seasonality and trends. To mend these gaps, we propose the STL-2DTCDN to deal with long-term multivariate time series forecasting tasks. STL-2DTCDN use the STL^32^ to decompose original series into three different subseries. The primary contributions of this paper can be summarized as follows:

1. We present the STL-2DTCDN for long-term multivariate time series forecasting. It follows a hybrid structure similar to most recent studies but incorporates enhanced component methods. The STL-2DTCDN achieves the state-of-the-art performance on six practical multivariate time series datasets, with a significant improvement in prediction accuracy.
2. Different from canonical TCN designed for single time series, we present a 2-dimensional temporal convolution dense network (2DTCDN) for multivariate time series forecasting. The 2DTCDN, employing 2D convolutional kernels, casual convolution, dilated convolution, and a dense layer, making it highly effective at capturing complex interdependencies among various time series in multivariate time series.
3. To enhance the model's ability in capturing seasonal and trend features, we integrate STL as the processing method. The original time sequence data is decomposed into different subseries with STL: seasonal, trend, and residual. These derived subseries can effectively illustrate the seasonal and trend characteristics inhered in the primitive data.

## Related works

### Methods for time series forecasting

Given the time series prediction tasks hold paramount significance in real world applications, numerous methods have been meticulously developed. Many traditional time series prediction models begin with statistical methods^33,34^. ARIMA^7^ adopts the Markov process to constructs the autoregressive model for iterative sequential forecasting. Nevertheless, an autoregressive process is incapable when handling complex sequence with nonlinearity and non-stationarity. As the evolution of neural networks over the past decades, RNN^9^ has been specially designed for processing sequential data. To address the challenge of gradient vanishing, many studies propose various modifications of RNN such as LSTM^12^ and GRU^13^. TCN^14^ is a neural network that employs dilated causal 1D convolution layers tailored for 1D data. However, as the convolution kernel with limited receptive field, the vanilla TCN is unable to explore interdependencies among various time series in multivariate time series data.

As a single model may fall short in learning complex features, some works pay attention to integrate various methods to one framework and present many ensemble models for time series forecasting. Ensemble models have been used in many practical applications successfully, such as traffic forecasting^17^, financial forecasting^18^, and energy management^35,36^. Moreover, ensemble learning methods hold robustness and reliability. Therefore, ensemble models have advantages in the field of medical applications^37,38^.

With the Transformer's impact on natural language processing and computer vision in recent years, there has been a surge in discussions, adaptations, and applications of Transformer-based solutions in time series forecasting. As for network modifications, various adaptations of Transformer for time series can be summarized into two levels: architectural and modular^39^. Some approaches, including Informer^24^, Autoformer^25^, and FEDformer^26^, modify the vanilla positional encoding of Transformer to leverage timestamps of time series and redesign the attention calculation methods to reduce complexity. Besides adapting individual modules within Transformer for time series modeling, approaches like Informer^24^ and Pyraformer^40^ aim to reconfigure Transformer at the architectural level. Notably, recent studies by Zeng et al.^30^ and Das et al.^31^ have demonstrated that linear models possess a strong capability for temporal relation extraction. In many cases, these linear models outperform most Transformer-based models in the area of time series forecasting.

### Decomposition of time series

Entangled temporal features in multivariate time series forecasting present significant challenges when it comes to effectively exploring local and long-range dependencies among time points and variables^41^. Many methods identify temporal dependencies with entangled temporal patterns, but they often can hardly fully leverage the inherent complex features of time series data, such as seasonality and trends. Therefore, various studies adopt time series decomposition to analyze time series. These decomposition methods can be divided into three categories: frequency domain decomposition, time domain decomposition, and time–frequency domain decomposition. The Fourier transform (FT)^25,26^ is a widely-recognized frequency domain decomposition technique in time series analysis. FT and its modifications can transform an original sequence from time domain to frequency domain, but they ignore trends shifts of time series. The STL is an important time domain decomposition method, which can effectively decompose a time series data into three distinct subseries. These three components represent different underlying categories of patterns that exhibit higher predictability. Wavelet transform (WT)^42,43^ and empirical wavelet transformation (EWT)^44,45^ are time–frequency domain decomposition methods that are particularly well-suited for the analysis of non-stationary series, as they can provide enhanced local time–frequency information.

## Methodology

We begin by introducing the formulation representation of multivariate time series forecasting. Subsequently, all involved components and the architecture of the proposed STL-2DTCDN model are presented. Finally, we detail the objective function and the evaluation metric employed for model training.

### Problem statement

The formulation is articulated as follows: Given one historical time data denoted as ${Y}_{1:L}={\left\{{y}_{1}^{t},{y}_{2}^{t},...,{y}_{c}^{t}\right\}}_{t=1}^{L}$ for $t=1$ to *L*, where *L* is the fixed look-back window, *c* (*c* > 1) is the number of variates, and ${y}_{i}^{t}$ denotes the value of the ${i}_{th}$ variate at the ${t}_{th}$ time. The multivariate time series prediction tasks aiming to figure out the predicted series ${\widehat{Y}}_{L+1:L+H}={\left\{{\widehat{y}}_{1}^{t},{\widehat{y}}_{2}^{t},...,{\widehat{y}}_{c}^{t}\right\}}_{t=L+1}^{L+H}$, where *c* (*c* > 1) denotes the number of variates, ${\widehat{y}}_{i}^{t}$ is the predicted result of the ${i}_{th}$ variate at the time step $t$, and *H* (*H* > 1) denotes the number of forecasting time steps. The ground truth for the time period from *L* + 1 to *L* + *H* is denoted as ${Y}_{L+1:L+H}={\left\{{y}_{1}^{t},{y}_{2}^{t},...,{y}_{c}^{t}\right\}}_{t=L+1}^{L+H}$. Long-term multivariate time series forecasting aims to forecast $\widehat{Y}$ with a larger value of *H* ($H\gg 1$).

Multi-step forecasting can be categorized into two types: iterated multi-step (IMS)^46^ forecasting and direct multi-step (DMS)^47^ forecasting. IMS forecasting iteratively predicts each time step, but it suffers from error accumulation effects. Compared to IMS forecasting, DMS forecasting can directly learn all prediction results at once. Consequently, DMS forecasting can outperform IMS forecasting in long-term forecasting tasks.

### Seasonal-trend decomposition based on loess (STL)

STL is an effective approach that can decompose an original time sequence data into three different subseries, which can be formulated as:

1 $${Y}_{t}={T}_{t}+{S}_{t}+{R}_{t}$$

where $t=\mathrm{1,2},...,n$ represents time steps, ${Y}_{t}$ denotes the original time series data, ${T}_{t}$, ${S}_{t}$, and ${R}_{t}$ represents the trend, seasonal, and residual components, respectively. In contrast to traditional decomposition methods, STL provides much more robust components for effectively decomposing time series sequence, especially in the presence of outliers. STL methodology consists of two iterative processes, known as the inner loop and the outer loop. Seasonal smoothing and trend smoothing during a single iteration are conducted in the inner loop, updating both the seasonal and trend components. Suppose ${{T}_{t}}^{k}$ and ${{S}_{t}}^{k}$ are the trend and seasonal components at the end of the ${k}_{th}$ iteration of the inner loop, respectively. Steps of computing ${{T}_{t}}^{k+1}$ and ${{S}_{t}}^{k+1}$ for the ${(k+1)}_{th}$ inner loop are detailed as follows:

*Step 1*: Detrending. Computing the detrend series ${Y}_{t}^{detrend}={Y}_{t}-{T}_{t}^{k}$. If there is a missing ${Y}_{t}$ at a time step, then the ${Y}_{t}^{detrend}$ of that time step is also missing;

*Step 2*: Seasonal smoothing. Smoothing the ${Y}_{t}^{detrend}$ with a smoother using Loess to figure out the initial seasonal component ${\widehat{S}}_{t}^{k+1}$;

*Step 3*: Filtering with low-pass. Processing ${\widehat{S}}_{t}^{k+1}$ with a filter with low-pass and a subsequent using Loess to figure out any residual trend component ${{\widehat{T}}_{t}}^{k+1}$;

*Step 4*: Detrending. The seasonal elements ${S}_{t}^{k+1}$ of the ${(k+1)}_{th}$ inner loop is calculated by ${\widehat{S}}_{t}^{k+1}-{{\widehat{T}}_{t}}^{k+1}$;

*Step 5*: Deseasonalizing. Subtract the seasonal elements from the original sequence ${Y}_{t}$ to get the deseasonalized time series ${Y}_{t}^{detrend}={Y}_{t}-{S}_{t}^{k+1}$;

*Step 6*: Trend smoothing. The trend component ${{T}_{t}}^{k+1}$ is obtained by smoothing ${Y}_{t}^{detrend}$ the with a Loess smoother.

After finishing the inner loop, the initial sequence is decomposed into the trend elements and the seasonal elements, the residual elements are calculated by in the outer loop: ${R}_{t}^{k+1}={Y}_{t}-{T}_{t}^{k+1}-{S}_{t}^{k+1}$.

**Figure 1:**
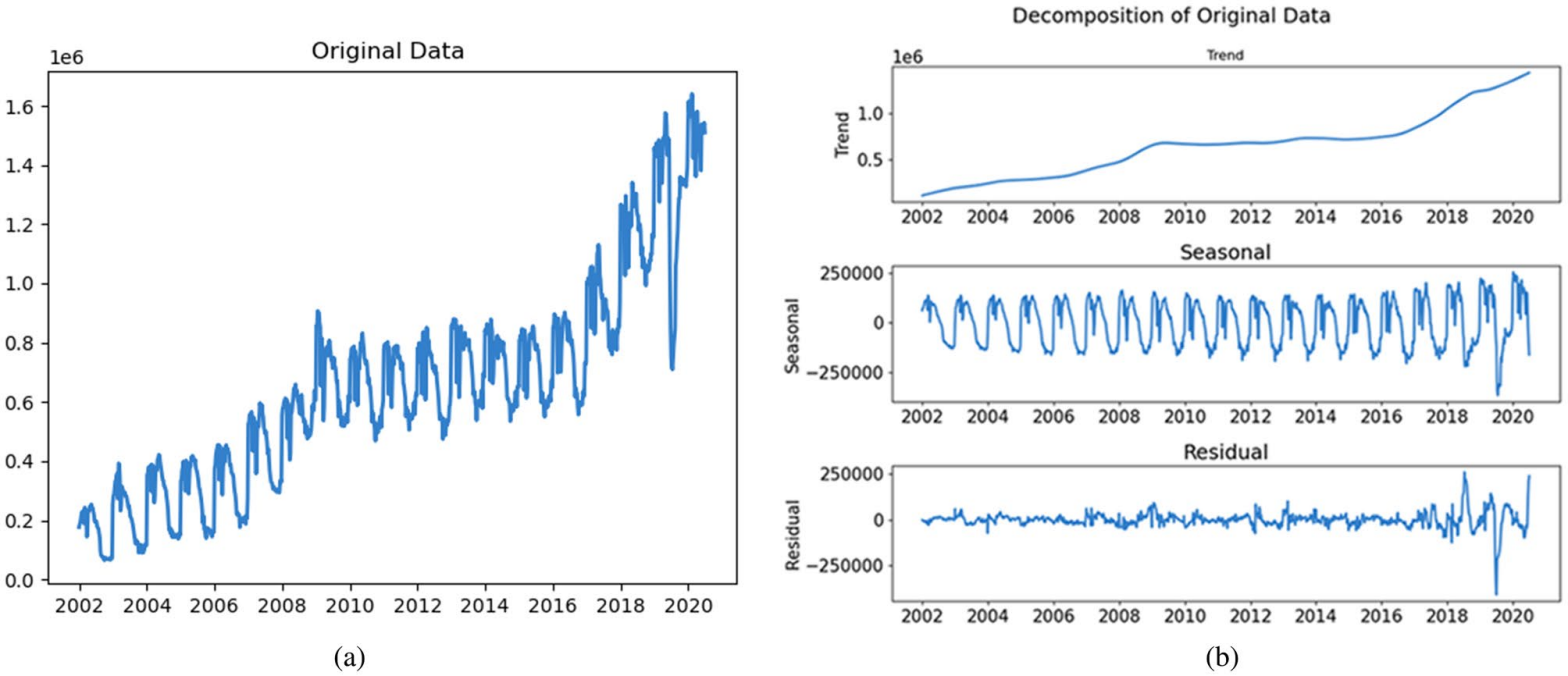
The decomposition results of STL. (**a**) Original data. (**b**) Decomposition of Original Data.

The parameters of STL were explored in previous experiments^41^. In this study, we set relevant parameters refer to the recommended defaults. Figure 1 presents the decomposition results of STL with the default parameter values, using data from the Centers for Disease Control and Prevention of the United States.

### 2-Dimensional temporal convolution dense network (2DTCDN)

**Figure 2:**
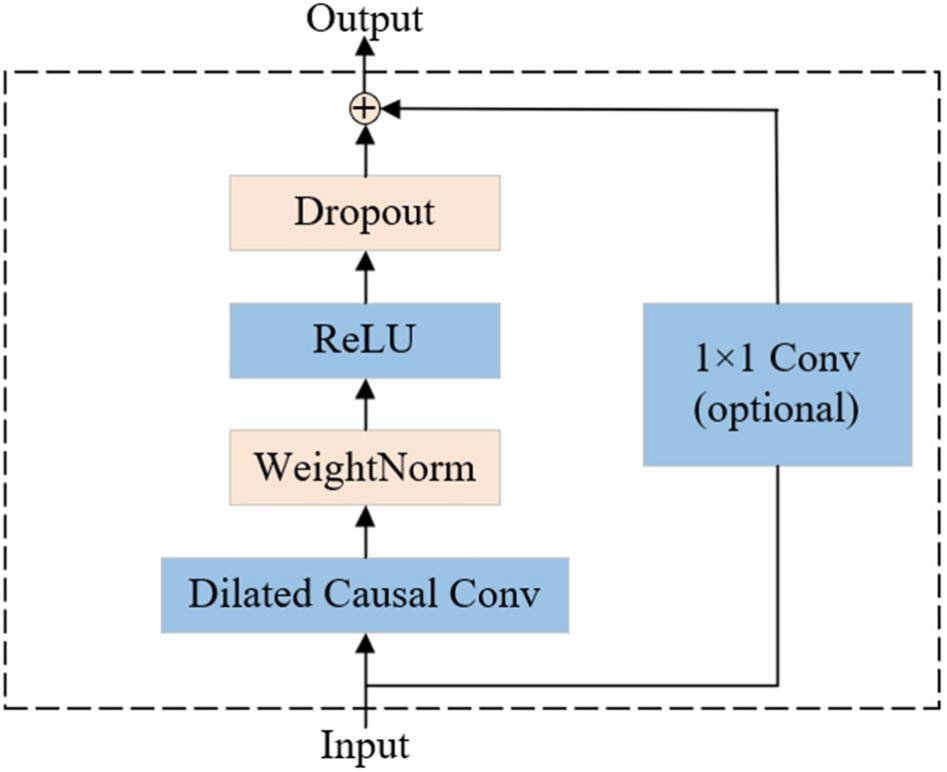
The architecture of TCN.

TCN is an effective approach proposed for modeling long sequence. Different from traditional RNN, TCN leverage the concept of CNN to explore complex dependencies in time sequences. The TCN architecture is presented in the Fig. 2, which consists of various layers, and an optional 1 × 1 convolution. Notably, dilated causal convolutions are used in TCN to increase the receptive field, enabling the capture of features at different time scales in time series data. For one 1-D sequence $X\in {R}^{M}$, and the filter ${K}_{d}$ with dilation rate $d$, the operation of the dilated causal convolution is defined as

2 $$\widehat{X}\left(t\right)={\sum }_{\tau =1}^{l}X(t-(d\cdot \tau ))\cdot {K}_{d}(\tau )$$

where $\widehat{X}\left(t\right)$ is the ${t}_{th}$ element of the output processed by the dilated causal convolution, $X(t-(d\cdot \tau ))$ represents the ${(t-(d\cdot \tau ))}_{th}$ element of the input sequence $X$, ${K}_{d}(\tau )$ denotes the ${\tau }_{th}$ element of the filter, $l$ is the length of the filter.

**Figure 3:**
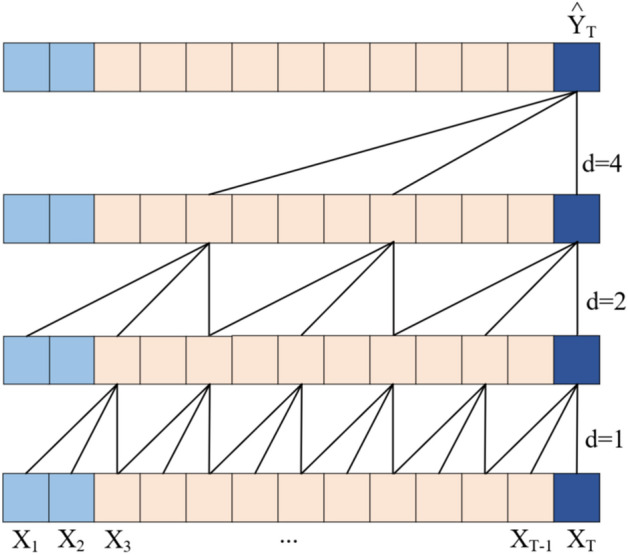
The dilated causal convolution.

**Figure 4:**
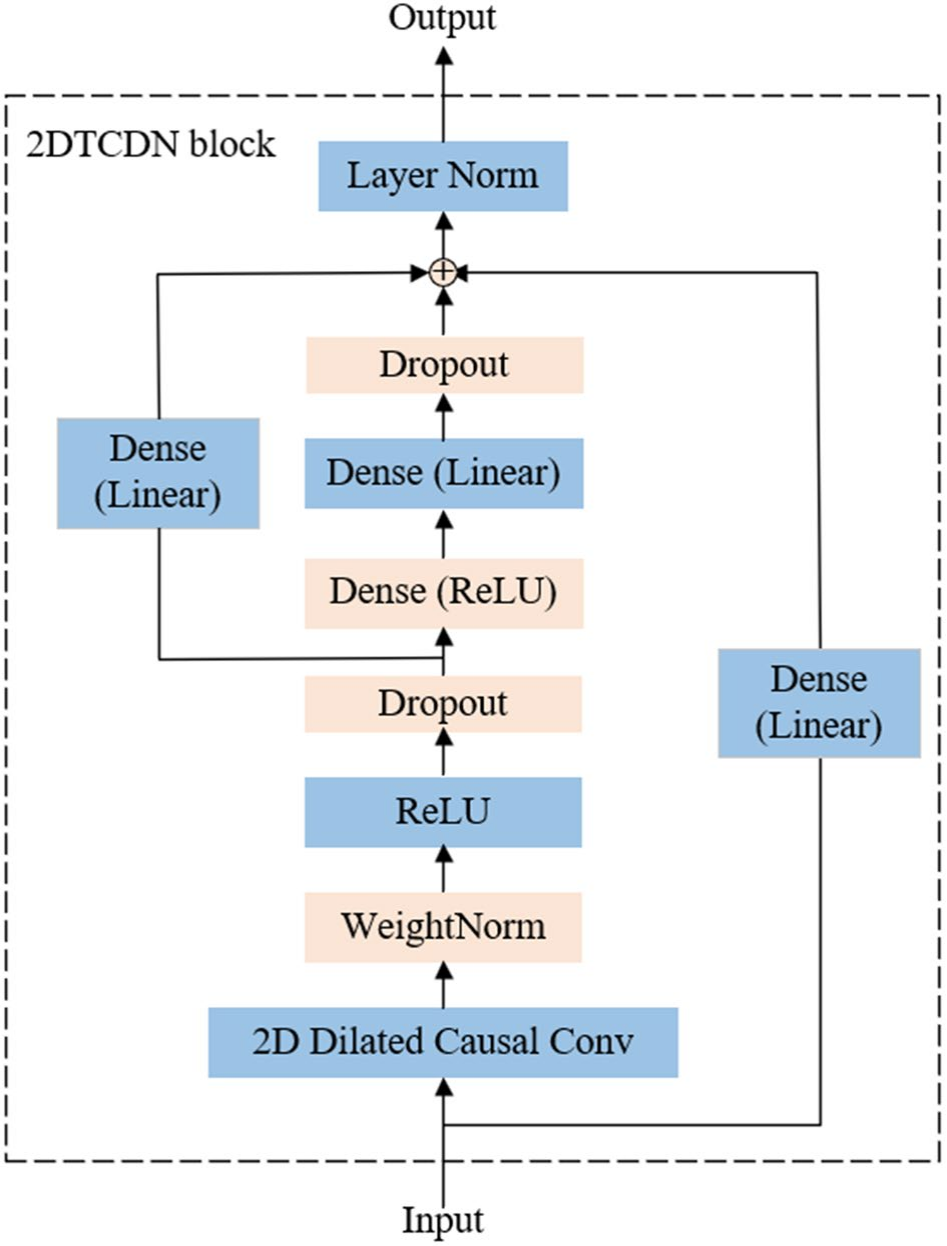
The architecture of 2DTCDN.

As illustrated in the Fig. 3, one framework of the dilated causal convolution with a filter size of *l* = 3 and dilation rate is set to *d* = 1, 2, 4. However, it's important to note that TCN's filter is one-dimensional (1D) and can only convolve along the time dimension of the time series. Consequently, TCN has limitations in capturing interdependencies among various time series in multivariate time series data. To better adapt TCN for multivariate time series forecasting tasks, we make some adjustments to the vanilla TCN and introduce the 2DTCDN. Figure 4 shows the architecture of 2DTCDN.

Causal convolution is an important concept, which limits that the output at time *t* is influenced by elements no later than *t*, ensuring that the future cannot influence the past. This concept is called information leakage, which is crucial in time series forecasting and it was initially proposed more than 30 years ago by Waibel et al.^48^. To maintain consistent dimensionality with the input layer and enable convolutions, zero padding is applied in the hidden layers. However, since we are using 2D convolutional kernels in the proposed 2DTCDN, the padding method and convolution process differ from that of the 1D dilated causal convolution. For one 2D sequence $X\in {R}^{M\times N}$, and the 2D filter ${K}_{d}$ with dilation rate d, the operation of the 2D dilated causal convolution is formulated as

3 $$\widehat{X}\left(i,j\right)=\sum\limits_{m=1}^{h}\sum\limits _{n}^{w}X(i-(m-1)\cdot d,j-(n-1)\cdot d)\cdot {K}_{d}(m,n)$$

where $\widehat{X}\left(i,j\right)$ is the ${(i,j)}_{th}$ element of the output processed by 2D dilated causal convolution, $X(i-(m-1)\cdot d,j-(n-1)\cdot d)$ represents the ${(i-(m-1)\cdot d,j-(n-1)\cdot d)}_{th}$ element of the input 2D matrix $X$, ${K}_{d}(m,n)$ denotes the ${(m,n)}_{th}$ element of the 2D filter, $h$ and $w$ respectively denote the height and width of the 2D filter.

**Figure 5:**
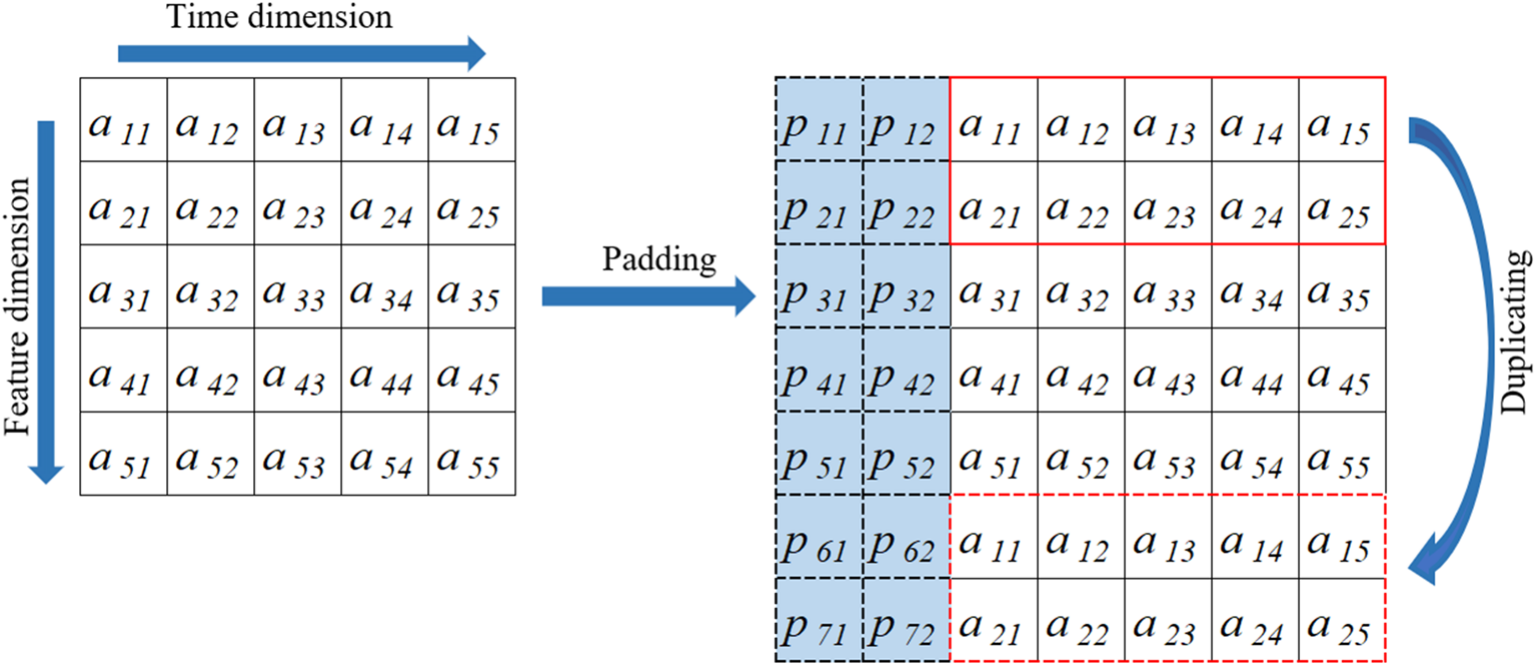
The padding method of 2DTCDN.

Figure 5 presents the padding of 2DTCDN, the kernel size is set to 3 × 3, dilation is set to 1. The padding of in the time dimension is similar to the 1D causal convolution, with the padding length calculated as $\left( {filter\_size {-} 1} \right) \times dilation$. In the feature dimension, the first '*padding length*' features are duplicated and placed after the last feature to serve as padding data.

**Figure 6:**
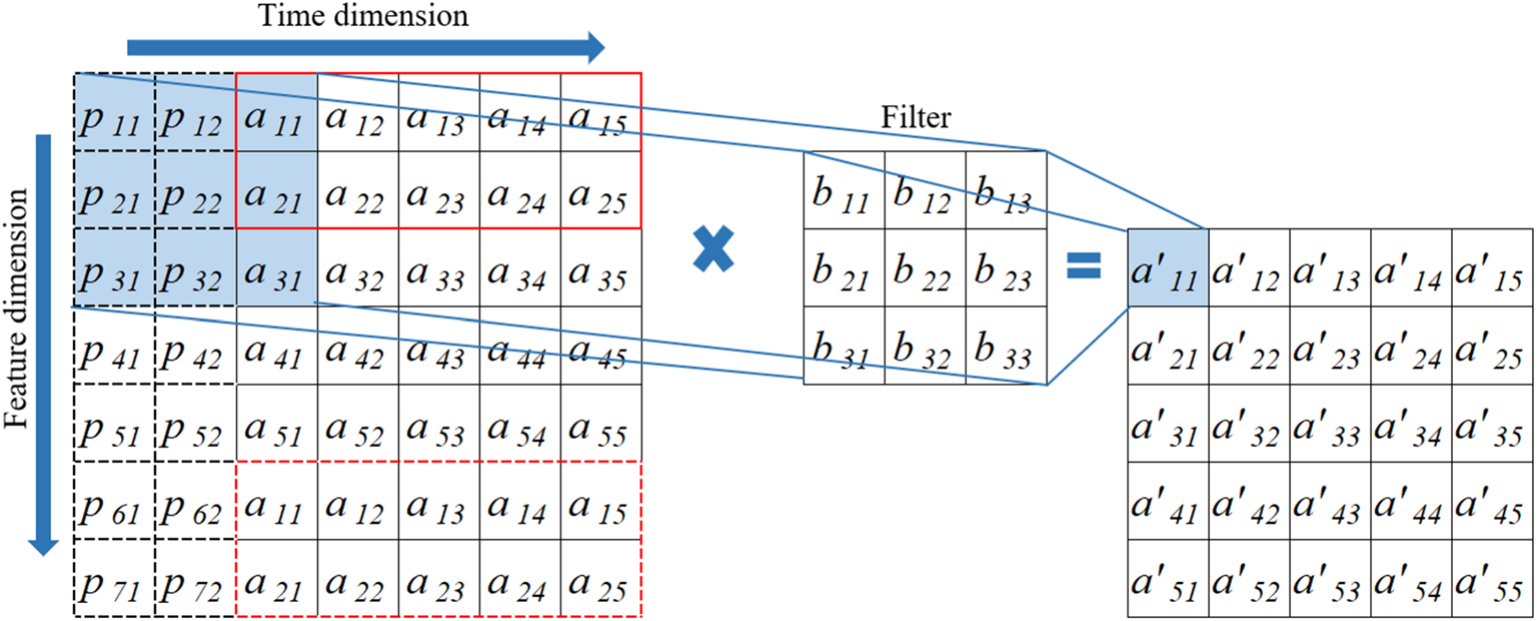
A dilated causal convolution process of 2DTCDN.

Dilated convolution is a variant of traditional convolutional operations used in deep learning^49^. In a standard convolution, a filter slides over the input data with a fixed stride, and each weight in the filter interacts with a neighboring input pixel. Different from the standard convolution, dilated convolution introduces gaps between the weights of the filter, allowing it to capture information from a broader receptive field while retaining the original resolution. For example, Fig. 6 illustrates a 2D dilated causal convolution process, the filter size is set to 3 × 3, dilation and stride are both set to 1.

### Residual connections

Residual connections are a fundamental architectural component in deep neural networks proposed by He et al.^50^. These connections are employed to mitigate the vanishing gradient. The main idea behind residual connections is to add the skip connection between different layers, creating a shortcut path for the gradient during backpropagation. We adopt the dense layer as the residual connection in the proposed 2DTCDN.

### Dense layer

Recent studies by Zeng et al.^30^ and Das et al.^31^ have demonstrated the remarkable capabilities of simple linear models in time series prediction tasks. Essentially, only one simple one-layer linear model can explore complex interdependencies within the sequence data effectively, allowing the neural network to explore intricate features that are important for the task. In our proposed 2DTCDN, we integrate the residual block of TiDE with 2D dilated causal convolution.

### Overview of the STL-2DTCDN framework

**Figure 7:**
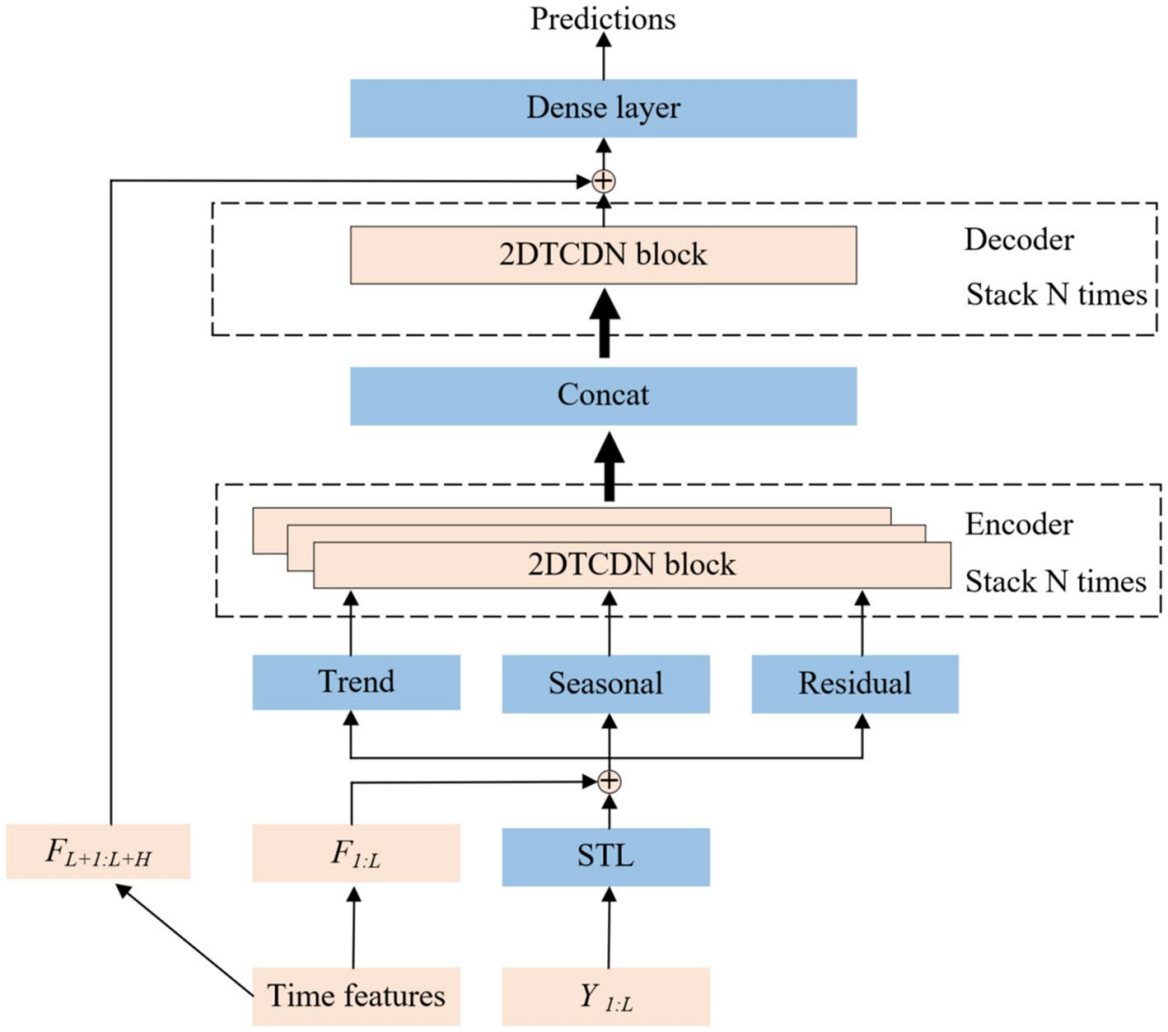
The framework of the STL-2DTCDN.

Figure 7 illustrates the entire framework of the STL-2DTCDN. We use ${F}_{t}$ to denote the time features at time step *t*. These time features include the holidays, the day of week, or other specific to a particular time step. The time series are first decomposed into three sub-series: trend (${T}_{t}$), seasonal (${S}_{t}$), and residual (${R}_{t}$) using STL. Subsequently, these three sub-series, concatenated with time features, are separately processed by an encoder architecture with the 2DTCDN block. Next, the processed sub-series are concatenated to form the input data of the next decoder architecture. Finally, outcomes of the decoder layer concatenated with time features are delivered to a dense layer to generate the predictions.

### Objective function

The squared error is a loss function frequently employed in time series prediction tasks. It evaluates the squared differences between the real values and predicted values. The optimization objective is formulated as:

4 $$Loss=\underset{t\in Train}{min}\sum\limits_{j=1}^{c}\sum\limits_{i=1}^{H}{\Vert {y}_{t+i}^{j}-{\widehat{y}}_{t+i}^{j}\Vert }_{F}^{2}$$

where $Train$ denotes a set of training time steps, $t$ represents the time step, $H$ represents the horizon of prediction, $c$ is the number of subseries, ${\parallel \parallel }_{F}$ is the Frobenius norm.

### Evaluation metrics

Mean absolute error (MAE) and mean square error (MSE) are adopted as the evaluation metric to assess the performance models. they are defined as:

5 $$MAE=\frac{1}{c\times H}\sum\limits_{j=1}^{c}\sum\limits_{i=1}^{H}\left|{y}_{t+i}^{j}-{\widehat{y}}_{t+i}^{j}\right|$$

6 $$MSE=\frac{1}{c\times H}\sum\limits_{j=1}^{c}\sum\limits_{i=1}^{H}{\left({y}_{t+i}^{j}-{\widehat{y}}_{t+i}^{j}\right)}^{2}$$

where $t$ represents the time step, $H$ indicates the horizon of prediction, and $c$ is the number of subseries.

## Experiments

### Datasets

The proposed STL-2DTCDN is tested on six datasets. All datasets are split into three segments in a chronological order: training, validation, and test sets, with a split ratio of 7:1:2 for Traffic and Electricity. ETT dataset are split with the ratio of 6:2:2, as recommended by Informer^24^ and Autoformer^25^. Table 1 presents statistical information of the datasets.

**Table 1:** Statistical information of six datasets.

Datasets	ECL	ETTh1	ETTh2	ETTm1	ETTm2	Traffic
Features	321	7	7	7	7	862
Timesteps	26,304	17,420	17,420	69,680	69,680	17,544
Frequency	1 Hour	1 Hour	1 Hour	15 Minute	15 Minute	1 Hour

• ETT (Electricity Transformer Temperature)^24^ including two datasets in 1-h-level (ETTh1, ETTh2) and two datasets in 15-min-level (ETTm1, ETTm2). Each dataset consists of seven electricity transformer attributes.

• Traffic^30^ collects data from the California.

• Electricity (ECL)^30^ describes the electricity consumption (Kwh) of 321 clients.

### Methods for comparison

At present, deep learning-based methods are the predominant approach in time series forecasting. We select six baseline methods for comparison with the STL-2DTCDN. These selected baseline methods including three categories: the TCN^14^ model, the Transformer-based methods (Informer^24^, Autoformer^25^, PatchTST/64^27^, and FEDformer^26^), and the linear models (DLinear^30^and TiDE^31^). TCN is designed for processing sequence data, and the adoption of causal convolution enhances its capacity in exploring dependencies of long-term. Transformer-based methods have made great success in time series forecasting tasks recently. In addition, linear models have been demonstrated that they can achieved promising results in various forecasting tasks.

The TiDE conducts experiments with the fixed look-back window 720 for all prediction lengths. Other compared models set the look-back windows as recommended. The results of baseline methods are reported from TiDE^31^ and PatchTST^27^.

### Experimental settings

The proposed STL-2DTCDN is trained using the L2 loss function and optimized with the ADAM^51^, initialized with a learning rate of 10^–4^. The look-back window for prediction lengths {96, 192, 336, 720} is all set to 720, following the TiDE. The batch size is set to 32 for training and experiments are repeated five times. Experiments are conducted using two NVIDIA GeForce RTX 2080 Ti GPUs, with the implementation in PyTorch. Table 2 presents the range of involved hyper-parameters. We tune these hyper-parameters by leveraging the rolling validation error on the validation dataset.

**Table 2:** Range of hyper-parameters.

Parameter	Range
Number of Encoder	[1, 2, 3]
Number of Decoder	[1, 2, 3]
Kernel size (time dimension)	[6, 12, 24, 48]
Kernel size (feature dimension)	[1, 2, 3, 4] or [4, 8, 12, 16]
Hidden size	[256, 512, 1024]
Time features hidden size	[16, 32, 64, 128]

Since the size of the look-back window is significantly different from the number of subseries for all datasets, the two dimensions of the convolution kernel are designed separately for the time and feature dimension. Specifically, considering the varying number of subseries in different datasets, such as ECL and Traffic with a larger number of time series, we utilize larger parameters [4, 8, 12, 16] in the feature dimension, while for datasets with fewer time series like ETT datasets, smaller parameters [1, 2, 3, 4] are employed in the feature dimension. Table 3 reports the chosen hyper-parameters for six datasets.

**Table 3:** Selected hyper-parameters for six datasets.

Datasets	ETTh1	ETTh2	ETTm1	ETTm2	ECL	Traffic
Encoder layers	2	2	2	2	2	2
Decoder layers	1	1	1	1	1	1
Kernel size (time dimension)	24	24	6	6	24	24
Kernel size (feature dimension)	3	3	3	3	12	16
Hidden size	512	512	512	512	512	512
Time features hidden size	64	32	64	128	128	64

### Experimental results

MAE and MSE of the proposed STL-2DTCDN and compared methods on six practical datasets are shown in Table 4. Each row in the table corresponds to a comparison of results within a specific window horizon, and each column represents the results of a particular model in all cases. The values that highlighted in bold are best results.

**Table 4:** Results of long-term multivariate time series forecasting on six datasets.

Models	STL-2DTCDN		TiDE		PatchTST/64		DLinear		FEDformer		Autoformer		Informer		TCN	
Metric	MSE	MAE	MSE	MAE	MSE	MAE	MSE	MAE	MSE	MAE	MSE	MAE	MSE	MAE	MSE	MAE
Traffic																
96	**0.305**	**0.242**	0.336	0.253	0.360	0.249	0.410	0.282	0.576	0.359	0.597	0.371	0.733	0.410	1.532	0.821
192	**0.317**	**0.256**	0.346	0.257	0.379	0.256	0.423	0.287	0.610	0.380	0.607	0.382	0.777	0.435	1.550	0.826
336	**0.324**	**0.246**	0.355	0.260	0.392	0.264	0.436	0.296	0.608	0.375	0.623	0.387	0.776	0.434	1.556	0.834
720	**0.347**	**0.267**	0.386	0.273	0.432	0.286	0.466	0.315	0.621	0.375	0.639	0.395	0.827	0.466	1.589	0.840
ECL																
96	0.129	0.230	0.132	0.229	**0.129**	**0.222**	0.140	0.237	0.186	0.302	0.196	0.313	0.304	0.393	1.103	0.902
192	**0.141**	**0.238**	0.147	0.243	0.147	0.240	0.153	0.249	0.197	0.311	0.211	0.324	0.327	0.417	1.113	0.898
336	**0.152**	**0.256**	0.161	0.261	0.163	0.259	0.169	0.267	0.213	0.328	0.214	0.327	0.333	0.422	1.167	0.915
720	**0.169**	**0.287**	0.196	0.294	0.197	0.290	0.203	0.301	0.233	0.344	0.236	0.342	0.351	0.427	1.334	0.935
ETTh1																
96	0.376	0.404	**0.375**	**0.398**	0.379	0.401	0.375	0.399	0.376	0.415	0.435	0.446	0.941	0.769	1.534	1.079
192	**0.401**	0.427	0.412	0.422	0.413	0.429	0.412	**0.420**	0.423	0.446	0.456	0.457	1.007	0.786	1.566	1.087
336	**0.412**	**0.426**	0.435	0.433	0.435	0.436	0.439	0.443	0.444	0.462	0.486	0.487	1.038	0.784	1.614	1.077
720	**0.426**	0.477	0.454	0.465	0.446	**0.464**	0.472	0.490	0.469	0.492	0.515	0.517	1.144	0.857	1.657	1.102
ETTh2																
96	**0.260**	**0.331**	0.270	0.336	0.274	0.337	0.289	0.353	0.332	0.374	0.332	0.386	1.549	0.952	1.876	1.345
192	**0.314**	**0.371**	0.332	0.380	0.338	0.376	0.383	0.418	0.407	0.446	0.426	0.434	3.792	1.542	1.547	1.752
336	**0.337**	**0.395**	0.360	0.407	0.363	0.397	0.448	0.465	0.400	0.447	0.477	0.479	4.215	1.642	1.791	1.749
720	**0.390**	**0.426**	0.419	0.451	0.393	0.430	0.605	0.551	0.412	0.469	0.453	0.490	3.656	1.619	2.501	3.622
ETTm1																
96	**0.287**	**0.325**	0.306	0.349	0.293	0.346	0.299	0.343	0.326	0.390	0.510	0.492	0.626	0.560	1.869	1.125
192	**0.315**	0.346	0.335	0.366	**0.333**	0.370	0.335	0.365	0.365	0.415	0.514	0.495	0.725	0.619	1.453	1.790
336	**0.347**	**0.368**	0.364	0.384	0.369	0.392	0.369	0.386	0.392	0.425	0.510	0.492	1.005	0.741	1.647	1.676
720	**0.408**	0.416	0.413	**0.413**	0.416	0.420	0.425	0.421	0.446	0.458	0.527	0.493	1.133	0.854	1.827	2.807
ETTm2																
96	0.164	0.253	**0.161**	**0.251**	0.166	0.256	0.167	0.260	0.180	0.271	0.205	0.293	0.355	0.462	1.633	1.258
192	**0.198**	**0.270**	0.215	0.289	0.223	0.296	0.224	0.303	0.252	0.318	0.278	0.336	0.595	0.586	1.996	1.385
336	**0.256**	**0.311**	0.267	0.326	0.274	0.329	0.281	0.342	0.324	0.364	0.343	0.379	1.270	0.871	1.872	1.221
720	**0.341**	**0.379**	0.352	0.383	0.362	0.385	0.397	0.421	0.410	0.420	0.414	0.419	3.001	1.267	2.003	1.438
Count	**38**		**5**		**4**		**1**		**0**		**0**		**0**		**0**	

Since all models are trained with the squared error, let's concentrate on the column of MSE column for comparisons. From Table 4, we can observe that: (1) The STL-2DTCDN can achieve the best results in most cases (as indicated by the count of the best results in the last row). (2) The STL-2DTCDN shows better performance than TiDE, and the MSE decreases by 3.2% (at 96), 5.7% (at 192), 5.8% (at 336), 6.9% (at 720) in average. The longer the prediction horizon, the better STL-2DTCDN performs. This suggests that STL-2DTCDN is more suitable for long-term forecasting. (3) Our proposed model achieves significantly better results than TiDE in large datasets for long-term forecasting. The MSE decreases by 10.1% (at 720) and 13.8% (at 720) for the Traffic dataset and the Electricity dataset, respectively. However, for four ETT datasets with the prediction length of 720, the STL-2DTCDN achieves only a 4.4% decrease in MSE on average compared to TiDE. We believe that this is because the Traffic and Electricity datasets have a significantly larger number of time series compared to four ETT datasets. Consequently, the 2DTCDN can utilize a larger kernel size in the feature dimension to better explore interdependencies among different time series.

**Figure 8:**
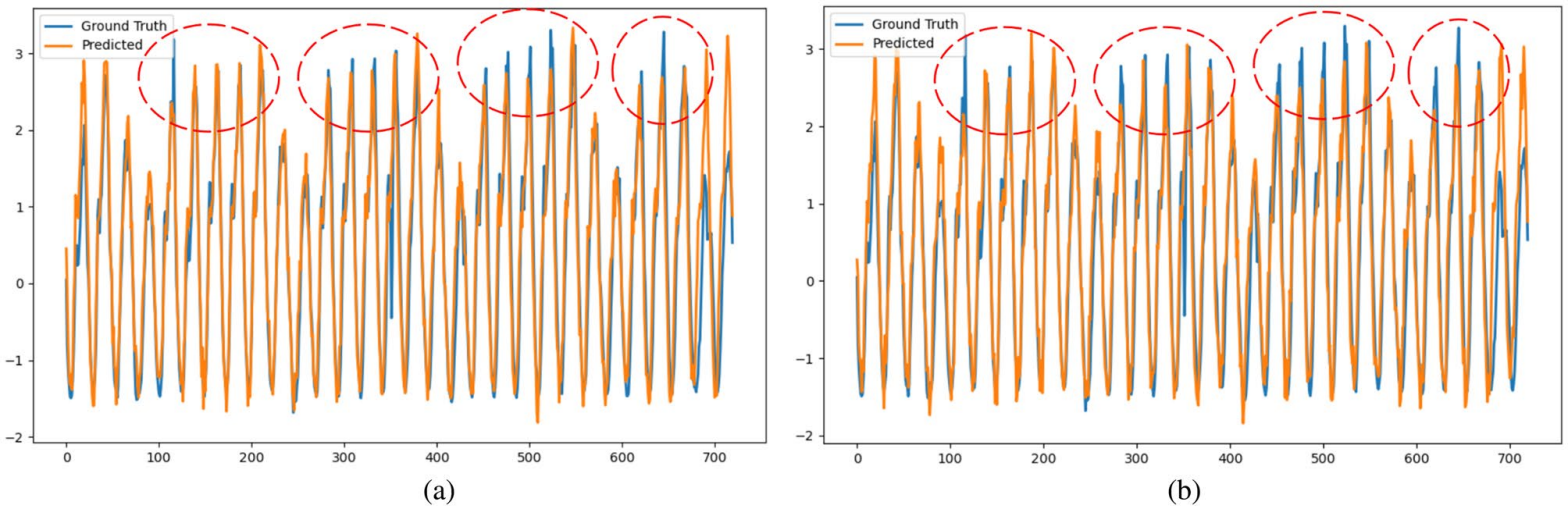
Comparison of the STL-2DTCDN and TiDE on the Traffic dataset (at 720). (**a**) STL-2DTCDN. (**b**) TiDE.

**Figure 9:**
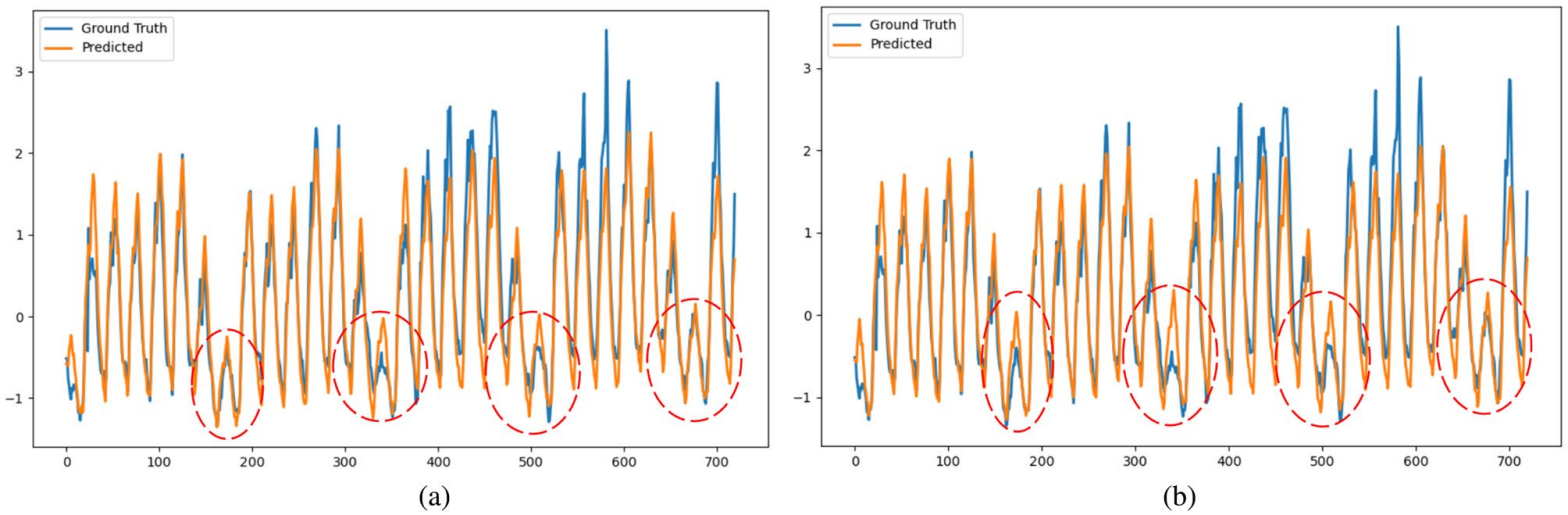
Comparison of the STL-2DTCDN and TiDE on the Electricity dataset (at 720). (**a**) STL-2DTCDN; (**b**) TiDE.

Figures 8 and 9 present the comparison of forecasting results calculated by STL-2DTCDN and TiDE with the ground truth for the Traffic and Electricity datasets. From these figures, we observe that the STL-2DTCDN performs better in capturing the temporal repeating patterns and fitting the trend of the curve. This indicates that the STL-2DTCDN effectively captures the temporal characteristics of the data sequences, including seasonality and trends.

### Ablation studies

The contribution of each involved component of STL-2DTCDN is figured out by ablation studies. Specifically, each component is removed in turn from the STL-2DTCDN, and we evaluate the performance of each sub-framework consists of the remaining components. Each sub-framework is detailed as follows:

- Re/STL: Remove the STL from the originally proposed STL-2DTCDN.
- Re/2DTCDN: Remove the 2DTCDN from the originally proposed STL-2DTCDN.
- Re/Time features: Remove the Time features from the originally proposed STL-2DTCDN.
- STL-2DTCDN → STL-TCN: 2DTCDN is replaced with a vanilla TCN.

Table 5 presents the performance of the original STL-2DTCDN and sub-frameworks by removing each component. It can be observed from the Table 5 that the combination of STL, 2DTCDN, and Time features delivers the most precise forecasts in different datasets, and the removal of any single component leads to a decline in performance. Furthermore, we also substitute the 2DTCDN with a standard TCN, and the forecasting results demonstrate that 2DTCDN can achieve better performance in long-term multivariate prediction tasks.

**Table 5:** Ablation study on different components of STL-2DTCDN (at 720).

Datasets	Traffic	ECL	ETTh2
STL-2DTCDN			
MSE	0.347	0.169	0.390
MAE	0.267	0.287	0.426
Re/STL			
MSE	0.461	0.235	0.511
MAE	0.379	0.336	0.576
Re/2DTCDN			
MSE	0.895	0.369	1.534
MAE	0.571	0.487	1.361
Re/Time features			
MSE	0.492	0.234	0.453
MAE	0.387	0.358	0.497
STL-2DTCDN → STL-TCN			
MSE	0.615	0.254	0.934
MAE	0.402	0.399	0.762

## Conclusions

We design a STL-2DTCDN model for long-term multivariate time series forecasting in this paper. STL-2DTCDN utilizes STL to decompose the original time sequence into three subseries. Time features is used to add additional covariates to the model. Furthermore, we adapt the vanilla TCN and introduce the 2DTCDN for long-term multivariate time series forecasting. Compared to various Transformer-based methods and linear models, the STL-2DTCDN exhibits strong capabilities in capturing various temporal patterns and exploring complex interdependencies between different related subseries for long-term multivariate time series forecasting. In the next stage, we will concentrate on: (1) interpreting the model outputs and understanding how a deep neural network achieves its forecasting results; (2) exploring alternative approaches to enhance the capability of capturing temporal patterns and exploring complex interdependencies inhered in multivariate time series.

## Data Availability

All datasets used can be accessed from the corresponding author on request.
